# Infrapopliteal angioplasty using a combined angiosomal reperfusion strategy

**DOI:** 10.1371/journal.pone.0172023

**Published:** 2017-02-15

**Authors:** G. K. Ambler, A. L. Stimpson, B. G. Wardle, D. C. Bosanquet, U. K. Hanif, S. Germain, C. Chick, N. Goyal, C. P. Twine

**Affiliations:** 1 Division of Population Medicine, Cardiff University, 3rd Floor Neuadd Meirionnydd, Heath Park, Cardiff, United Kingdom; 2 South East Wales Vascular Network, Royal Gwent Hospital, Cardiff Road, Newport, United Kingdom; Universidade de Mogi das Cruzes, BRAZIL

## Abstract

**Introduction:**

Infra-popliteal angioplasty continues to be widely performed with minimal evidence to guide practice. Endovascular device selection is contentious and there is even uncertainty over which artery to treat for optimum reperfusion. Direct reperfusion (DR) targets the artery supplying the ischaemic tissue. Indirect reperfusion (IR) targets an artery supplying collaterals to the ischaemic area. Our unit practice for the last eight years has been to attempt to open all tibial arteries at the time of angioplasty. When successful, this results in both direct and indirect; or combined reperfusion (CR). The aim was to review the outcomes of CR and compare them with DR or IR alone.

**Methods:**

An eight year retrospective review from a single unit of all infra-popliteal angioplasties was undertaken. Wound healing, limb salvage, amputation-free and overall survival data as well as re-intervention rates were captured for all patients. Subgroup analysis for diabetics was undertaken. Kaplan Meier curves are presented for survival outcomes. All odds and hazard ratios (HR) and p values were corrected for bias from confounders using multivariate analysis.

**Results:**

250 procedures were performed: 22 (9%) were CR; 115 (46%) DR and 113 (45%) IR. Amputation-free survival (HR 0.504, p = 0.039) and re-intervention and amputation-free survival (HR 0.414, p = 0.005) were significantly improved in patients undergoing CR compared to IR. Wound healing was similarly affected by reperfusion strategy (OR = 0.35, p = 0.047). Effects of CR over IR were similar when only diabetic patients were considered.

**Conclusions:**

Combined revascularisation can only be achieved in approximately 10% of patients. However, when successful, it results in significant improvements in wound healing and amputation-free survival over simple indirect reperfusion techniques.

## Introduction

Infrapopliteal angioplasty continues to be debated in the literature. Not only is the choice of endovascular device contentious [1], but there is even uncertainty over which artery to treat for optimum reperfusion in the context of tissue loss [2]. The main problem is a lack of good evidence; meta-analyses are still drawing on a small number of low volume cohort studies to make recommendations, and randomised trials do not exist [1, 2]. The ongoing BASIL-2 [3] and BEST-CLI [4] trials addresses the question of whether an endovascular first or open bypass first strategy is optimal in patients with infrapopliteal disease. However, the question of which endovascular strategy to adopt is still not addressed.

The debate over the optimum strategy for infra-popliteal reperfusion is based on the angiosome model. Direct reperfusion (DR) targets the artery supplying the ischaemic tissue. Indirect reperfusion (IR) targets an artery supplying collaterals to the ischaemic area: most often the peroneal for tissue loss in the foot [5–7]. The indirect approach has the advantage of preserving the direct artery, so if damage occurs at the time of angioplasty the surgical target remains intact. However a recent meta-analysis found that IR was associated with poorer wound healing and limb salvage outcomes when compared to DR [2].

Our unit practice for the last eight years has been to open every possible tibial artery at the time of angioplasty. When successful, this leads to combined (both direct and indirect) reperfusion (CR). This strategy is contentious, and potential drawbacks include added length of procedure, risk of thrombosis, occlusion of a successfully reperfused vessel, and cost. Only one series has previously been published on this technique [8] so it could not be included in the most up to date meta-analysis on angiosomal reperfusion [2]. The aim was therefore to review the outcomes of a CR strategy in terms of wound healing; amputation rates; re-interventions and overall survival, and to compare them with DR or IR alone.

## Methods

A retrospective review of consecutive patients undergoing infra-popliteal angioplasties for critical ischaemia (Rutherford 4–6) over an eight year period (January 2009 to December 2016) at a single center was performed. The tibial arteries were defined as anterior tibial artery, tibioperoneal trunk, peroneal artery, posterior tibial artery and pedal arteries. Basic demographics, comorbidities, pre-operative blood tests, smoking status and available components of the Society for Vascular Surgery (SVS) Threatened Limb Classification System (Wound, Ischemia, Foot infection: WIFi classification) [9] were recorded to allow correction of results for confounding factors. Patients were excluded if they had received previous infra-popliteal angioplasty to the same limb, unless all wounds on the treated leg had been documented as healed at least 12 months prior to the subsequent procedure. The project was registered and approved by Research and Development at Aneurin Bevan University Health board: St Woolos Hospital, 131 Stow Hill, Newport NP20 4SZ (email: ABB.R&D@wales.nhs.uk). Data were anonymised during analysis.

### Intervention

All patients were assessed by a consultant vascular surgeon and reviewed at a peripheral vascular multidisciplinary meeting (MDM). Duplex, Magnetic Resonance (MR) or Computerised Tomography (CT) angiography was performed prior to angioplasty. Our experience of MR and CT is that it does not give good enough images to plan distal bypasses so a formal angiogram was undertaken for patients being considered for distal bypass. Functionally suitable patients with occlusive disease reconstituting in the tibial arteries with a good distal target artery were offered bypass first. Patients deemed unfit for bypass or with an artery which the MDM felt would not sustain bypass were offered angioplasty first. Multilevel stenotic rather than occlusive disease was treated with angioplasty first.

All patients underwent intervention by one of two interventional radiologists (NG or CC) using the same technique. In all cases CR was attempted preferentially; however DR or IR was performed by default when this was technically impossible. 3000iu intra-arterial heparin was administered prior to the procedure as standard. Non-drug eluting angioplasty balloons were used. Bailout stenting was not routine in the tibial arteries. Patients were routinely rediscussed at MDM and followed up in a consultant led vascular surgery, diabetic foot or wound healing clinic. Antiplatelet agents (Aspirin 75mg or Clopidogrel 75mg) and statin therapy (usually atorvastatin 80mg) were prescribed as routine pre and post procedure. If wounds failed to heal or the limb declined symptomatically, arterial imaging was repeated and the limb treated again if restenosis had occurred. If there was residual stenosis or occlusion in one vessel but another vessel was successfully treated this was defined by the successful vessel.

### Outcomes

The primary outcome measures were wound healing, amputation-free survival and limb salvage. Wound healing was defined as full epithelialisation. For patients with hospital records not capturing a fully healed wound we contacted the General Practitioner (GP) and/or tissue viability service for the date of healing. If the patient died of another cause during follow up and the wound was still under review this was classified as not healed. Amputation with a non-healed wound was also truncated as not healed.

Our group’s recent meta-analysis of DR versus IR of infra-popliteal arteries [2] suggested that DR had an odds ratio of 0.34 (95% CI 0.23–0.51) for successful wound healing over IR. If the true odds ratio is taken conservatively as the upper limit of the confidence interval (0.51), the proportion of CR or DR:IR is approximately 1:1 as in many previous studies, and approximately one third of wounds heal with revascularization, then simulation studies suggest that a sample size of 200 limbs would exceed 90% to detect a difference of this magnitude at the 5% level.

Limb salvage was defined as an intact limb with no below or above knee amputation. Patients with healed minor amputations were considered to have a salvaged limb. If the patient died of another cause with an intact limb this was classified as successful limb salvage truncated at the time of death. The exception to this was if the limb had an unhealed wound as above. Amputation-free survival was defined as alive with an intact limb and no above the ankle amputation. Outpatients with wounds were seen in a multidisciplinary wound clinic with a specific wound healing specialist. Planned amputations had the decision made from this clinic. Overall survival and reintervention and amputation-free survival defined (as in the BEST-CLI trial [4]) as survival free from above-ankle amputation of the index limb or re-intervention were assessed as secondary outcomes. Re-intervention was defined as repeat infra-popliteal angioplasty or distal bypass surgery of the treated limb. All data were collected from the health board electronic record system linking GP and Hospital record data which has been running for over 10 years. If this was lacking the paper notes were examined and/or GP surgeries contacted as above. This is linked into the Office for National Statistics for survival data from the whole of the United Kingdom.

Procedures were deemed technically successful if blood flow was restored with no significant residual stenosis in the treated vessel(s). If CR was attempted but only IR or DR was achieved this was classified accordingly. Renal failure was defined according to Chronic Kidney Disease stage. Angiosomes were as defined by Taylor and Palmer, modified slightly in Iida et al 2012 [10]. Rest pain in the toes without tissue loss was considered to be the anterior tibial or posterior tibial angiosomes for DR.

### Statistical analysis

Statistical analysis appropriate for non-parametric data was used. Grouped data were expressed as a median (range). Groups were compared with the Kruskal-Wallis and Fisher’s exact tests for continuous and categorical data respectively. Wound healing was assessed both in terms of absolute healing rates, with confounder correction performed using multivariate logistic regression modelling, and also in terms of time to healing using multivariate Cox regression analysis to correct for confounders. Cumulative survival was calculated by the life table method of Kaplan and Meier. Kaplan Meier curves are presented for survival outcomes. All odds ratios (OR), hazard ratios (HR) and p values were corrected to control for bias from confounding variables using multivariate regression analysis (either logistic regression or Cox regression depending on the outcome). Forward conditional regression analysis with minimisation of Akaike’s Information criterion (AIC) was used to assess whether confounder adjustment was required for age, gender, pre-procedural haemoglobin, white blood cell count, albumin, creatinine, comorbidities, smoking status and the SVS wound and foot infection scores. Data analysis was carried out with the R statistics package version 3.3.1 together with the built-in ‘survival’ package version 2.40–1.

## Results

Two hundred and fifty consecutive limbs with critical ischemia underwent infra-popliteal angioplasty. Twenty-two (9%) were CR; 115 (46%) DR and 113 (45%) IR. Two limbs were counted twice as a result of deterioration of a limb following previous successful angioplasty with fully healed ulcers, in one case 26 months after the initial intervention, in the other case 32 months after initial intervention (22 and 29 months after initial ulcer healing respectively). Twenty-seven patients underwent infra-popliteal angioplasty on both legs, so there were a total of 221 patients treated. Based on the power calculations described above in the Methods, this should be enough to detect differences in healing rates as large as those seen in previous studies.

Demographic details, comorbidities known to affect outcomes after tibial angioplasty and indication for intervention including available components of the WIFi classification are shown in Table 1. There was a higher proportion of female patients in the DR group, and there was a small but significant difference in the prevalence of diabetes subtypes between the groups. Age differences approached significance. Ankle brachial pressure indices and toe pressures were not routinely used as part of clinical care within our unit, and these were recorded prior to only 19 of the 250 procedures, prohibiting meaningful comparisons, so are not recorded in Table 1.

**Table 1:** Patient demographics.

		All (patients n = 221)	CR (limbs n = 22)	DR (limbs n = 115)	IR (limbs n = 113)	P
*Sex (M:F)*		156:65	18:4	71:44	89:24	0.01
*Median Age (range)*		75 (36–99)	72 (36–95)	73 (47–99)	77 (41–94)	0.05
*Hypertension (%)*		79	73	76	85	0.14
*Ischaemic Heart Disease (%)*		42	36	42	49	0.43
*Diabetes*	*Diabetes Any*	70	59	75	73	0.32
	*Type 1*	10	27	12	8	0.02
	*Type 2*	59	32	63	65	0.04
*Smoker (%)*	*Current*	26	23	21	31	0.27
	*Ex*	38	45	37	41	0.69
*CKD stage (%)*	*3*	21	9	21	23	0.37
	*4*	6	5	8	5	0.85
	*5*	7	9	8	7	0.88
*SVS Wound*	*0*	12	9	10	11	0.77
*Score (%)*	*1*	49	45	56	57	0.13
*N = 250*	*2*	32	45	27	27	0.16
	*3*	7	0	7	7	0.44
*SVS Foot*	*0*	33	27	30	37	0.50
*Infection Score*	*1*	25	18	25	26	0.82
*(%, n = 250)*	*2*	26	36	27	23	0.39
	*3*	16	18	17	14	0.76
*Indication (%,*	*Tissue Loss*	87	86	90	85	0.58
*n = 250)*	*Rest pain alone*	13	14	10	15	0.43
	*Threatened graft*	2	0	1	3	0.56

CR = Combined direct and indirect reperfusion; DR = Direct reperfusion; IR = Indirect reperfusion; IHD = Ischaemic Heart Disease. Smoking status could not be determined for eight patients (6 treated with direct revascularization and 2 with indirect revascularization).

In one hundred and seventeen of the procedures (47%), angioplasty of another artery was performed at the same time as a tibial artery. Three procedures (1%) included iliac angioplasty, 78 (31%) included superficial femoral angioplasty and 74 (30%) included popliteal angioplasty. Two-hundred and eight (83%) of procedures were considered successful by the treating interventionist in restoring in-line flow to the ankle.

Only four (2%) patients proceeded to bypass. Three patients (1.3%) showed signs of thrombosis, which was successfully cleared by a combination of suction, thrombolysis or repeat angioplasty. Thirty-five limbs (14%) required repeat revascularization (3 surgical bypass, 32 angioplasty) within 12 months of the initial intervention, and over the entire study period, 59 subsequent procedures (55 angioplasties and 4 surgical bypasses) were performed on the 248 limbs to maintain limb perfusion. Thirty-five limbs underwent 1 re-intervention, nine underwent 2 re-interventions and two underwent 3 re-interventions.

### Wound healing

Overall, 36% of wounds healed without above the ankle amputation, 55% after CR, 38% after DR and 32% after IR. Twenty-nine-percent proceeded to major limb amputation, 18% died with a chronic wound and 17% were alive with a chronic wound when the study ended. Uncorrected analysis suggested that between-group differences in healing were significant (p = 0.003). After confounder adjustment using multivariate logistic regression modelling, the difference between CR and IR persisted (OR = 0.35, 95% CI 0.12–0.98, p = 0.047), though differences between CR and DR (OR = 0.51, 95% CI 0.18–1.40, p = 0.19), and between IR and the combination of CR or DR (OR = 0.62, 95% CI 0.35–1.10, p = 0.11) were not significant. Group comparisons were carried out between CR, DR and IR independently. There was no difference in wound healing times between the CR, DR and IR groups (Table 2).

**Table 2:** Infrapopliteal angioplasty outcomes by reperfusion strategy.

All procedures n = 250*	**CR vs. DR**	**CR vs. IR**	**CR and DR vs. IR**
Wound Healing rate	HR 1.08, p = 0.832	HR 0.751, p = 0.428	HR 1.41, p = 0.155
Limb salvage	HR 0.688, p = 0.404	HR 0.676, p = 0.387	HR 0.912, p = 0.709
Amputation free survival	HR 0.578, p = 0.103	HR 0.504, p = 0.039	HR 0.795, p = 0.159
Overall Survival	HR 0.516, p = 0.131	HR 0.431, p = 0.055	HR 0.768, p = 0.168
Reintervention and amputation free survival	HR 0.433, p = 0.009	HR 0.414, p = 0.005	HR 0.798, p = 0.140
*Diabetics only n = 181*			
Wound Healing rate	HR 1.14, p = 0.758	HR 0.758, p = 0.541	HR 0.683, p = 0.192
Limb salvage	HR 0.402, p = 0.110	HR 0.485. p = 0.214	HR 0.935, p = 0.808
Amputation free survival	HR 0.492, p = 0.082	HR 0.426. p = 0.037	HR 0.854, p = 0.399
Overall Survival	HR 0.795, p = 0.677	HR 0.568, p = 0.301	HR 0.698, p = 0.105
Reintervention and amputation free survival	HR 0.400, p = 0.017	HR 0.394, p = 0.015	HR 0.805, p = 0.219

*All hazard ratios and p-values are corrected for confounders found to be significant on stepwise minimisation of AIC. CR = Combined direct and indirect reperfusion; DR = Direct reperfusion; IR = Indirect reperfusion

### Limb salvage

**Fig 1:**
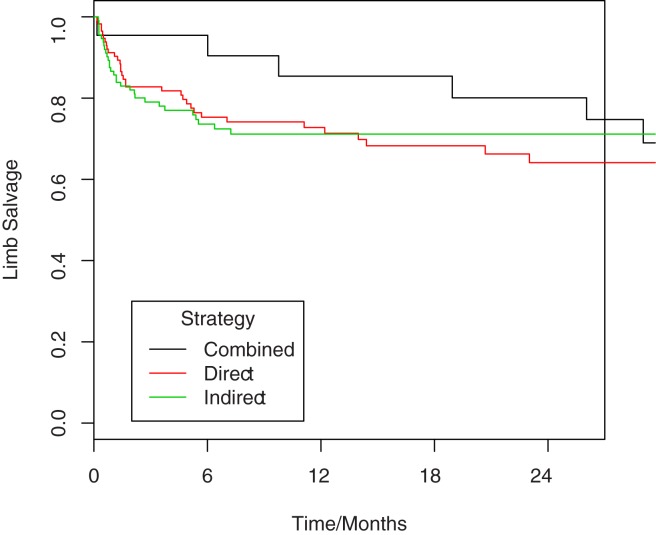
Kaplan-Meier analysis comparing overall limb salvage in patients undergoing combined approach angioplasty versus the indirect and direct approaches. HR 0.688, p = 0.404 for combined versus direct, HR 0.676, p = 0.387 for combined versus indirect. Numbers at risk are given in S1 Table.

Limb salvage was 73% at one year. In total, sixty-nine limbs were amputated, 55 below the knee and 14 above the knee. There was no difference in limb salvage by reperfusion strategy (Table 2, Fig 1, S1 Table).

### Amputation-free survival

**Fig 2:**
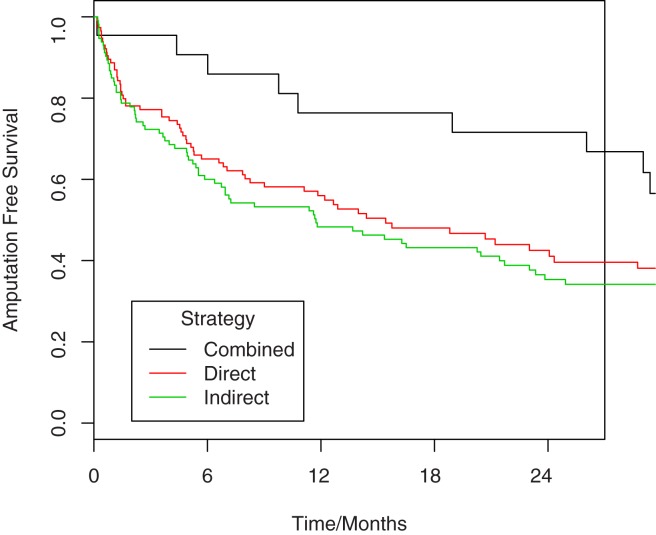
Kaplan-Meier analysis comparing amputation-free survival in patients undergoing combined approach angioplasty versus the indirect and direct approaches. HR 0.492, p = 0.082 for combined versus direct, HR 0.426, p = 0.037 for combined versus indirect. Numbers at risk are given in S1 Table.

Amputation-free survival was 54% at one year, falling to just 33% at 3 years. There was a difference in amputation-free survival by reperfusion strategy, with CR having a clear benefit over IR after adjustment for confounders (Table 2, Fig 2, S1 Table). This benefit was lost once the CR and DR groups were combined and compared to IR.

### Overall survival

Overall 1 year survival was 74%, falling to 49% at 3 years. There was no significant improvement in overall survival according to revascularisation strategy (Table 2).

### Reintervention and amputation free survival

**Fig 3:**
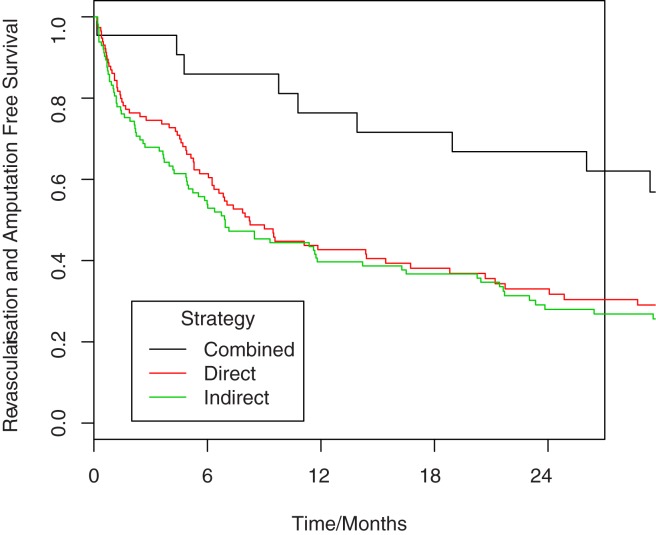
Kaplan-Meier analysis comparing reintervention and amputation free survival in patients undergoing combined approach angioplasty versus the indirect and direct approaches. HR 0.433, p = 0.009 for combined versus direct, HR 0.414, p = 0.005 for combined versus indirect. Numbers at risk are given in S2 Table.

Overall survival free from re-intervention or major limb amputation was 44% at 1 year, falling to just 25% at 3 years. Reintervention and amputation free survival was significantly better with a combined revascularisation strategy, compared to either direct (HR 0.433, p = 0.009) or indirect reperfusion (HR 0.414, p = 0.005) strategies (Table 2, Fig 3, S2 Table).

### Diabetic patients alone

One hundred and eighty-one limbs were treated in diabetic (type one and two) patients, accounting for 72% of the total cohort. Demographic details are shown in Table 3. The results in this group were only corrected for smoking. The results for wound healing, limb salvage and overall survival are shown in Table 2.

**Table 3:** Diabetic patient demographics.

		All (n = 181)	CA (n = 13)	DR (n = 86)	IR (n = 82)	P
Sex (M:F)		139:42	13:0	57:29	69:13	0.002
Median Age (range)		73 (36–94)	58 (36–82)	72 (47–94)	77 (43–93)	0.001
Hypertension (%)		83	77	79	88	0.24
Ischaemic Heart Disease (%)		51	46	50	52	0.89
*Smoker (%)*	Current	25	38	18	30	0.0001
	Ex	41	54	39	41	0.59
*CKD stage (%)*	3	24	8	26	24	0.40
	4	8	0	10	7	0.59
	5	10	8	10	10	1.0
*SVS Wound*	0	7	0	5	11	0.20
*Score (%)*	1	49	31	58	41	0.04
*N = 250*	2	37	69	30	39	0.02
	3	7	0	7	9	0.74
*SVS Foot*	0	25	8	22	32	0.13
*Infection Score*	1	25	15	27	24	0.75
*(%*, *n = 250)*	2	29	46	28	28	0.40
	3	20	31	23	16	0.28
*Indication (%*,	Tissue Loss	92	92	94	90	0.56
*n = 250)*	Rest pain alone	8	8	6	10	0.35
	Threatened graft	0	0	0	0	1.0

CR = Combined direct and indirect reperfusion; DR = Direct reperfusion; IR = Indirect reperfusion; IHD = Ischaemic heart disease. Smoking status could not be determined for six patients (4 treated with direct revascularization and 2 with indirect revascularization).

In contrast to what was found for the overall cohort, there were significant between-group differences in the subset of patients with diabetes in terms of sex ratios, ages, proportion of patients currently smoking and SVS Wound classification. All hazard ratios and p-values presented for the outcomes of interest have been corrected for these measured confounders using multivariate Cox regression modelling.

### Wound healing

Overall, 34% of wounds in diabetic patients healed without requiring above the ankle amputation. There was no difference in rates of wound healing between the CR, DR and IR groups (Table 2).

### Limb salvage

Overall limb salvage in diabetic patients was 71% at one year. There was no significant difference in limb salvage by reperfusion strategy (Table 2).

### Amputation-free survival

Overall amputation-free survival in diabetic patients was 50% at one year, falling to just 29% at 3 years. CR was significantly better than IR in diabetic patients, even after adjustment for measured confounders (HR 0.426, p = 0.037). This advantage disappeared when the CR group was combined with patients treated with a DR strategy (Table 2).

### Overall survival

Overall 1 year survival in diabetic patients was 70%, falling to 45% at 3 years. Revascularisation strategy did not significantly affect overall survival (Table 2).

### Reintervention and amputation free survival

Overall survival free from reintervention or major limb amputation was 40% at 1 year, falling to just 20% at 3 years in diabetic patients. Reintervention and amputation free survival was significantly better with a combined revascularisation strategy, compared to either direct (HR 0.400, p = 0.017) or indirect reperfusion (HR 0.394, p = 0.015) strategies (Table 2).

## Discussion

This study shows that CR has advantages over IR alone in the tibial arteries. While effects suggested that there was also an advantage over DR, this was statistically significant only in terms of improved reintervention and amputation free survival. Results were similar in the subset of diabetic patients.

These data represent the largest series examining attempted CR in the literature and will be useful to add to aggregated data in the future; we could not include CR in our group’s meta-analysis of tibial angioplasty but with this series it could be included in future analyses. We have corrected for bias as much as is possible, which has never been performed in previous published studies.

While we have made every effort to correct for known confounders, it is likely that unmeasured confounders persist, as is the case in all retrospective cohort series. The practitioners set out to perform CR so, in effect; DR or IR was a reflection of unsuccessful CR. The DR and IR only cohorts will therefore have, by definition, more severe arterial disease than the CR cohort. The IR cohort especially will have more severe disease and the outcomes appear to reflect this. Having acknowledged this, it is unknown how many patients undergoing DR from a different cohort would fail IR if it was attempted, and vice versa. This really means that CR was equivalent to DR in a limb where IR was also attempted then failed. This may mean that the results here would not apply to patients in whom DR or IR was intended as the outcome from the start of the procedure. This bias applies across the whole of the angiosome literature which led to a very low GRADE quality recommendation in the recent meta-analysis on this topic [2]. Although the cohort is comparatively large within the literature on infra-popliteal angioplasty, low patient numbers may lead to inadequate power for primary outcomes comparing CR and DR to IR. As there is little literature on CR, it was not possible to perform sample size calculations to estimate the power of the study to show differences between CR and DR or IR. Had differences between DR and IR been similar to those reported in previous studies, the study would have been adequately powered to detect this, however the differences we saw between the combination of DR and CR, and IR were much smaller than those reported previously, explaining our negative finding for this comparison. Importantly, CR takes longer than opening up one vessel and comes with the increased risk entailed in angioplasty of more than one tibial artery. It is also potentially more expensive as further angioplasty balloons may need to be opened.

The findings of this series are different from our meta-analysis of all other series in the literature, which suggested that DR is better than IR [2]. Wound healing and limb salvage rates in this series were similar to those meta-analysed [5, 8, 10–13]. Importantly, we could not analyse CR in our previous meta-analysis as there was only one published series in which it was performed [14]. That series found no advantage of CR compared to treating a single vessel, but their number of patients treated was less than half that presented here. They could also not fully examine DR vs. IR due to low patient numbers [14].

A previous large cohort in which multiple tibial arteries were treated found that the more arteries opened at the time of angioplasty the better the clinical result [15]. In that study the authors did not account for angiosome targeted reperfusion, opened all three tibial arteries in many patients and reported an 83% patency at one year [15]. Our findings disagree with this and it is likely that the good results in the former study were the result of selection bias; three tibial arteries are difficult to open in our patient population with critical ischaemia and our practice would have been to not treat the tibial arteries if all three were patent with minor stenotic disease only. This, and many other studies also treat claudicants [15–18] for whom we do not perform tibial angioplasty. Including claudicants would significantly improve limb salvage compared to our data, which means our comparable limb salvage rates may actually be slightly higher than previous cohorts.

Our results suggest that diabetic patients benefit as much from CR as non-diabetic patients. Previous pure diabetic cohorts have found benefit for DR but may have treated less severe disease because of the inclusion of claudicants [19]. The angiosome model may seems less relevant in diabetes because of the pattern of tibial disease being different from other patient populations; there is some evidence to support a different pattern of choke and collateral arteries [6, 20]. Our results do not support this view. There are also additional factors to confound results in diabetes such as infection, extent of tissue damage and blood glucose control which are known to affect wound healing. Our use of the wound and foot infection elements of the SVS WIFi classification to allow correction for these confounders should minimise the impact of these factors on the presented statistics.

The effect observed for amputation free survival is interesting. There is evidence that chronic ischaemia leads to a higher rate of ischaemic cardiovascular events and worse amputation free survival. This may explain some of the effect here, as may a higher mortality as a result of amputation in the IR group.

Post procedure we use a wound healing service run by a dedicated wound healing expert which means results may not be comparable to other areas in the UK where patients may be cared for by district nurses or non-specialists post procedure.

The other significant question this cohort cannot address is the value of tibial angioplasty in addition to angioplasty to the knee. Although outcomes did not differ between the group undergoing tibial angioplasty and tibial angioplasty in addition to an inflow procedure (data not shown), the numbers in the cohort are not large enough to confidently answer this question. This series, like all others published, also addresses radiological/surgical outcomes and does not examine quality of life or limb which are more important. A fully powered randomised trial or automatic data capture in a national database would be needed to explore this fully.

## Conclusion

A combined angiosomal reperfusion strategy shows superiority over indirect reperfusion alone in a cohort where combined reperfusion was attempted preferentially. The optimum reperfusion strategy during tibial angioplasty combining these results with previous meta-analysis, should therefore be to attempt to restore as much flow to the ischaemic area as possible. Indirect reperfusion still appears useful if a direct approach fails.

## Supporting Information

S1 Table Number at risk and amputation free survival at 6, 12, 18 and 24 months data for Fig 1. (DOCX)

S2 Table Number at risk and survival free from reintervention or amputation at 6, 12, 18 and 24 months data for Fig 2. (DOCX)
