# Supplementary material for: Infrapopliteal angioplasty using a combined angiosomal reperfusion strategy
Source: PLoS One. 2017 Feb 15;12(2):e0172023. doi: 10.1371/journal.pone.0172023 (PMC5310906; doi:10.1371/journal.pone.0172023)
Supplement: S1 Table — (DOCX) [file pone.0172023.s001.docx]

**S1 Table.** Number at risk for limb salvage and amputation free survival (AFS) at 6, 12, 18 and 24 months data for Figures 1 and 2.

|  | **Months** | **6** | **12** | **18** | **24** |
| --- | --- | --- | --- | --- | --- |
| **CR** | N at Risk | 19 | 16 | 16 | 15 |
|  | Limb salvage % | 96 | 85 | 85 | 80 |
|  | AFS % | 86 | 76 | 76 | 72 |
| **DR** | N at Risk | 68 | 51 | 37 | 29 |
|  | Limb salvage % | 75 | 73 | 68 | 64 |
|  | AFS % | 65 | 56 | 48 | 43 |
| **IR** | N at Risk | 63 | 49 | 42 | 30 |
|  | Limb salvage % | 74 | 71 | 71 | 71 |
|  | AFS % | 60 | 48 | 43 | 35 |
