# Supplementary material for: Infrapopliteal angioplasty using a combined angiosomal reperfusion strategy
Source: PLoS One. 2017 Feb 15;12(2):e0172023. doi: 10.1371/journal.pone.0172023 (PMC5310906; doi:10.1371/journal.pone.0172023)
Supplement: S2 Table — (DOCX) [file pone.0172023.s002.docx]

**S2 Table.** Number at risk and reintervention and amputation free survival (RAFS) at 6, 12, 18 and 24 months data for Figure 3.

|  | **Months** | **6** | **12** | **18** | **24** |
| --- | --- | --- | --- | --- | --- |
| **CR** | N at Risk | 18 | 16 | 15 | 14 |
|  | RAFS % | 86 | 76 | 72 | 67 |
| **DR** | N at Risk | 64 | 41 | 31 | 25 |
|  | RAFS % | 61 | 43 | 38 | 33 |
| **IR** | N at Risk | 57 | 42 | 37 | 25 |
|  | RAFS % | 54 | 40 | 37 | 28 |
